# Influence of an abscisic acid (ABA) seed coating on seed germination rate and timing of Bluebunch Wheatgrass

**DOI:** 10.1002/ece3.5212

**Published:** 2019-06-14

**Authors:** William C. Richardson, Turmandakh Badrakh, Bruce A. Roundy, Zackary T. Aanderud, Steven L. Petersen, Phil S. Allen, Dallin R. Whitaker, Matthew D. Madsen

**Affiliations:** ^1^ Department of Plant and Wildlife Sciences Brigham Young University Provo Utah

**Keywords:** germination delay, rangeland improvement, restoration, seed dormancy, seeding, thermal time, wet‐thermal accumulation model

## Abstract

Semi‐arid rangeland degradation is a reoccurring issue throughout the world. In the Great Basin of North America, seeds sown in the fall to restore degraded sagebrush (*Artemisia* spp.) steppe plant communities may experience high mortality in winter due to exposure of seedlings to freezing temperatures and other stressors. Delaying germination until early spring when conditions are more suitable for growth may increase survival. We evaluated the use of BioNik™ (Valent BioSciences LLC) abscisic acid (ABA) to delay germination of bluebunch wheatgrass (*Pseudoroegneria spicata*). Seed was either left untreated or coated at five separate rates of ABA ranging from 0.25 to 6.0 g 100 g^−1^ of seed. Seeds were incubated at five separate constant temperatures from 5 to 25°C. From the resultant germination data, we developed quadratic thermal accumulation models for each treatment and applied them to 4 years of historic soil moisture and temperature data across six sagebrush steppe sites to predict germination timing. Total germination percentage remained similar across all temperatures except at 25°C, where high ABA rates had slightly lower values. All ABA doses delayed germination, with the greatest delays at 5–10°C. For example, the time required for 50% of the seeds to germinate at 5°C was increased by 16–46 d, depending on the amount of ABA applied. Seed germination models predicted that the majority of untreated seed would germinate 5–11 weeks after a 15 October simulated planting date. In contrast, seeds treated with ABA were predicted to delay germination to late winter or early spring. These results indicate that ABA coatings may delay germination of fall planted seed until conditions are more suitable for plant survival and growth.

## 1 INTRODUCTION

Disturbance and interannual precipitation variability cause arid and semi‐arid rangelands across the world to be susceptible to a decline in environmental health (GLP, 2005; Reynolds et al., 2007; Zhao, Zhao, Gao, & Ho, 2017). The sagebrush (*Artemisia* spp.)‐steppe ecosystem in the Great Basin region of North America is an example of a degraded rangeland, which currently exists on only 56% of its historic range (Hardegree, Jones, Roundy, Shaw, & Monaco, 2016; Suring, Rowland, & Wisdom, 2005). Loss of the sagebrush steppe is commonly caused by invasion and dominance of annual grasses, which increases fire frequency and perpetuates weed dominance (Baker, 2006; Balch, Bradley, D'Antonio, & Gomez‐Dans, 2013; Dantonio & Vitousek, 1992). Cheatgrass (*Bromus tectorum* L.) is a dominant invasive annual weed that increases wildfire frequency by producing a dry, fine, continuous fuel layer early in the summer season (Bradley, Houghtonw, Mustard, & Hamburg, 2006; Dantonio & Vitousek, 1992; Germino, Chambers, & Brown, 2016). The dominance of cheatgrass also decreases organic carbon stored in the soil (Rau et al., 2011) and reduces the habitat of wildlife species such as sage‐grouse (*Centrocercus urophasianus*), that depend on the sagebrush steppe system (Knick et al., 2003). To reduce weed dominance and stabilize soils, native and introduced perennial species are typically seeded in fall after wildfires (Crawford et al., 2004; Madsen, Davies, Boyd, Kerby, & Svejcar, 2016; Richards, Chambers, & Ross, 1998). However, the success of native plant establishment from seed has been highly variable  (Hardegree et al., 2016).

Plant recruitment may be limited by ecological processes, such as freezing and thawing of the seedbed, development of physical soil crusts, and pathogen attack, during the first winter the seeds are sown (Hardegree et al., 2016; James, Svejcar, & Rinella, 2011). In the Great Basin, soil water recharge occurs in fall, winter, and spring (Roundy et al., 2014). Wildfires in sagebrush steppe generally occur during the hot and dry summer period. Burned rangelands are typically seeded in fall before the soils begin to freeze and before soil moisture is too high to operate planting equipment (Amaranthus & Perry, 1989; Beyers, 2004). James et al. (2011) and Boyd and James (2013) found that approximatly 80% of fall‐seeded perennial grasses, such as bluebunch wheatgrass (*Pseudoroegneria spicata* (Pursh) Á. Löve), germinated prior to winter but only a small percentage of the seeds sown (10%–15%) produced seedlings that emerged from the soil in the spring.

Seed dormancy may prevent seed germination until conditions are favorable for establishment and growth (Allen, Benech‐Arnold, Batlla, & Bradford, 2007; Baskin & Baskin, 2001; Finch‐Savage & Leubner‐Metzger, 2006). Dormancy mechanisms vary across species through adaptation to the prevailing environment (Baskin & Baskin, 2001, 2004; Finch‐Savage & Leubner‐Metzger, 2006). Physiological dormancy is the most abundant form of dormancy and varies in its depth from species that require several months of stratification before germination, to species that can germinate following brief after‐ripening in dry storage (Baskin & Baskin, 2005, 2001). Seed dormancy may be induced through the addition of the plant hormone abscisic acid (ABA) during seed maturation on the mother plant (Kucera, Cohn, & Leubner‐Metzger, 2005). Within the seed, ABA is a regulator of both the induction and maintenance of dormancy, which functions through a complex network of signaling pathways (Finch‐Savage & Leubner‐Metzger, 2006; Zhao et al., 2011). Within the seed, it is not necessarily the concentration of ABA that effects dormancy but its relation to the concentration of gibberellins (GA) (Dekkers & Bentsink, 2015; Finch‐Savage & Footitt, 2017; Finch‐Savage & Leubner‐Metzger, 2006; Lefebvre et al., 2006; LeonKloosterziel et al., 1996). When the ratio of ABA concentration to GA concentration is higher, seeds are more likely to maintain some dormancy (Duclos, Altobello, & Taylor, 2014; Kermode, 2005). Additionally, both the localization of ABA and the competency of cells to respond to the hormone play important roles in breaking seed dormancy (Finch‐Savage & Leubner‐Metzger, 2006).

Applications of ABA may have the potential to re‐induce seed dormancy to delay germination of fall‐sown seeds. The effects of ABA on seeds in laboratory and agricultural experiments show that ABA can delay germination when applied exogenously (Aroca, Alguacil, Vernieri, & Ruiz‐Lozano, 2008; Atia, Debez, Barhoumi, Smaoui, & Abdelly, 2009; Hussaina et al., 2015; Papenfus, Kulkarni, Stirk, Finnie, & Staden, 2013; Romagosa et al., 2001). However, no study has demonstrated the effect, ABA would have on seeds used for restoration efforts in rangeland settings. Research is needed to understand the rate ABA should be applied to sufficiently delay germination to at least late winter or early spring when temperatures are less likely to damage plant tissue (Pearce, 2001). Roundy and Madsen (2016) reported that freezing conditions in Great Basin sagebrush communities could last as long as 168 days (October to mid‐March).

Wet‐thermal accumulation models may offer the first step in determining if ABA application rates in a seed coating are sufficient to delay germination until after this freezing period. Germination timing of many nondormant seed populations is a function of temperature accumulation when seeds are imbibed (Rawlins, Roundy, Davis, & Egget, 2012). Wet‐thermal accumulation models predict the timing and rate of seed germination based on temperature, with progress toward germination accumulated when temperature and soil water potential are above a set threshold (Forcella, Arnold, Sanchez, & Ghersa, 2000; Vleeshouwers & Kropff, 2000). Rawlins, Roundy, Egget, and Cline (2012) found that wet‐thermal accumulation models that were applied to field soil moisture and temperature data could accurately predict germination timing with a 50%–95% accuracy, depending on the season the seeds were sown.

The objectives of this study were to: (a) assess the effect of ABA application rate, applied within a seed coating, on final germination percentage, seed germination timing, and the spread of when the seeds would germinate (synchrony) under different constant temperatures, (b) for each unique seed coating, create thermal accumulation models that express how germination timing changes with temperature, and (c) apply thermal accumulation models to field soil moisture and temperature data sets across the Great Basin to predict seed germination timing from simulated planting dates. We hypothesized that ABA seed coatings would delay seed germination, with the extent of delay a function of the rate of ABA applied. We also hypothesized that models would predict that ABA coatings could provide sufficient delay to allow seed germination to occur in spring.

## 2 MATERIALS AND METHODS

### 2.1 Seed coatings

We used “Anatone” bluebunch wheatgrass as the model species to investigate the effect of ABA rates on seed germination. Bluebunch wheatgrass is a common perennial bunchgrass throughout much of the Intermountain West, USA. This species provides quality forage for livestock and wildlife, helps suppress weeds, and is frequently seeded in restoration projects. Certified seed was purchased from Granite Seed and Erosion Control.

A formulation of S‐ABA was obtained from Valent BioSciences LLC, under the trade name BioNik™. Seven different rates of BioNik were applied to the seed at rates of 0.25, 0.5, 1.0, 1.5, 2.0, 4.0, and 6.0 g BioNik 100 g^−1^ seed (as formulation). A coated treatment without any ABA was not added to the study because preliminary laboratory trials showed no significant difference in germination time and total germination between untreated seed and coated seed (Badrakh, 2016).

Seeds were coated under a two‐step process in a 30‐cm rotary seed coater from Universal Coating Systems. We applied BioNik during the first step of the coating process in a dilution of Agrimer SCP binder (Ashland Inc.). In this first step, a total of 10 g of this mixture was applied to the seed. In the second step, 50 g of Agrimer SCP and 175 g of calcium carbonate powder (limestone) were added slowly to the seed to cover the BioNik coating and build up the size of the seed. The coated seed was then dried using a forced air dryer at 43°C (Brace Works Automation and Electric).

### 2.2 Germination experiment

In addition to the seven ABA treatments, untreated seeds (control) were included in the experiment. Each treatment was repeated seven times. In each replicate, 25 seeds were placed in 13 × 13 cm acrylic boxes (Pioneer Plastics) filled with 140 g of fine sand. Before planting, the sand was watered to field capacity. Seeds were placed on the surface of the sand, and the boxes were sealed to maintain moisture levels. The study was installed as a randomized complete block split‐plot design, with temperature comprising the split‐plot factor. We used a range of constant temperatures to germinate seeds (5, 10, 15, 20, 25°C). Seeds were placed in Precision Plant Growth Chambers (Thermo Fischer Scientific) to maintain the different temperatures, and each chamber was set to a 12‐hr light/12‐hr dark regime. Each block had one of each treatment and took up an entire shelf in the growth chamber. The number of germinated seeds was counted every 1–3 d. We defined germination as the extension of the radical 2 mm from the seed. Once germinated, seeds were removed from the boxes. After each counting, the blocks were randomly placed on a new shelf within the growth chamber.

From laboratory seed germination counts, we calculated several seed dormancy indices, final germination percentage, time to reach germination at 10% intervals from 10% to 90%, and germination synchrony. Final germination percentage was calculated as the ratio of the number of seeds germinated to the total number of seeds. Time to reach a certain germination percentage (T_x_, i.e., time to reach 10% germination is T_10_) was calculated as follows:

(1) $$T_{x}=\left[\left(\frac{t_{a}-t_{b}}{n_{a}-n_{b}}\right)\left(N-n_{b}\right)\right]+t_{b}$$

where: T_x_ is equal to time (days) to subpopulation germination, t_a_ is equal to the incubation day when subpopulation germination was reached, t_b_ is equal to the incubation day before subpopulation germination was reached, n_a_ is equal to the number of germinated seeds on the day that subpopulation germination was reached, n_b_ is equal to the number of germinated seeds on the day before subpopulation germination was reached, and N is equal to the number of germinated seeds equal to the targeted subpopulation (Rawlins, Roundy, Davis, et al., 2012). Germination synchrony was calculated by subtracting T_90_ from T_10_.

We created mixed models to determine the significance (*p* < 0.05) of ABA dose , incubation temperature, and their interactions (unless determined not to be significant) for final germination percentage, T_50_, and synchrony of germination. In the model, blocks were considered random, while incubation temperature and treatment were both considered fixed. We tested for differences in responses to ABA levels at the incubation temperatures of 5, 10, 15, 20, and 25°C using a Tukey pairwise comparison test (*p* < 0.05). The square root of T_50_ was used to normalize the data, but a transformation of total germination percentage and synchrony was not needed as indicated by viewing residuals.

### 2.3 Prediction of seed germination timing in the field

Using the program Auto Germ (Richardson, et al., 2018), linear and curvilinear regression was used to apply polynomial equations to the germination time data gathered from the experiment in the previous section. These equations, or wet‐thermal models, estimate germination rate (inverse of T_x_) in relation to incubation temperature. Germination rate was used instead of T_x_ to improve model accuracy (Rawlins, Roundy, Davis, et al., 2012). Models were created for all seed treatments, for each of the germination intervals described above. These models were then applied to historical soil temperature and water potential data from the Sagebrush Step Treatment and Evaluation Project (SageSTEP); Cline, Roundy, & Christensen, 2018a; Cline, Roundy, & Christensen, 2018b). We selected from the SageSTEP network six different sites to model seed germination timing, which were within Wyoming big sagebrush (*Artemisia tridentata* Nutt. ssp. *wyomingensis* Beetle & Young) communities that had all been treated with prescribed burns (Moses Coulee, WA, Saddle Mountain, WA, Hart Mountain, OR, Roberts, ID, Owyhee, NV, and Onaqui, UT) (McIver & Brunson, 2014). At each of the sites, hourly measurements were made at approximately 1–3 cm below the soil surface to estimate soil temperature using thermocouples and soil water potential using gypsum blocks (Delmhorst, Inc.). Gypsum block resistance was converted to MPa of water potential using standard calibration curves (Rawlins, Roundy, Egget, et al., 2012).

Estimates of seed germination timing were predicted at each site over four years (2011–2014) with the exception of the Moses Coulee site, which did not have enough data to be used for 2011. A simulated planting date of 15 October was set for modeling seed germination timing, which is a common time for land managers to initiate seeding projects in the Great Basin. In calculating seed germination timing, progress toward germination was determined for each individual hourly soil temperature data point starting at the planting date. Progress toward germination was calculated by dividing hourly soil temperature by the time to reach T_x_ at the temperature of that data point (determined using the regression models described above). In this model, thermal progress toward germination is accumulated only for hours when soil water potential is above −1.5 Ψ (Rawlins, Roundy, Egget, et al., 2012). This ratio, or progress toward germination, was then converted to a percentage and accumulated until 100% was reached. At that point, we determined that T_x_ was reached. The process was repeated for each thermal model created. From this data, we determined the time when the majority of seed for each treatment would germinate (i.e. month of the year when >50% of the population had germinated) and then averaged the data across all sites and years. Additionally, graphs were developed that show when each treatment of seed would reach each T_x_ interval for each site and simulated planting year.

## 3 RESULTS

### 3.1 Final germination percentage

**Figure 1:**
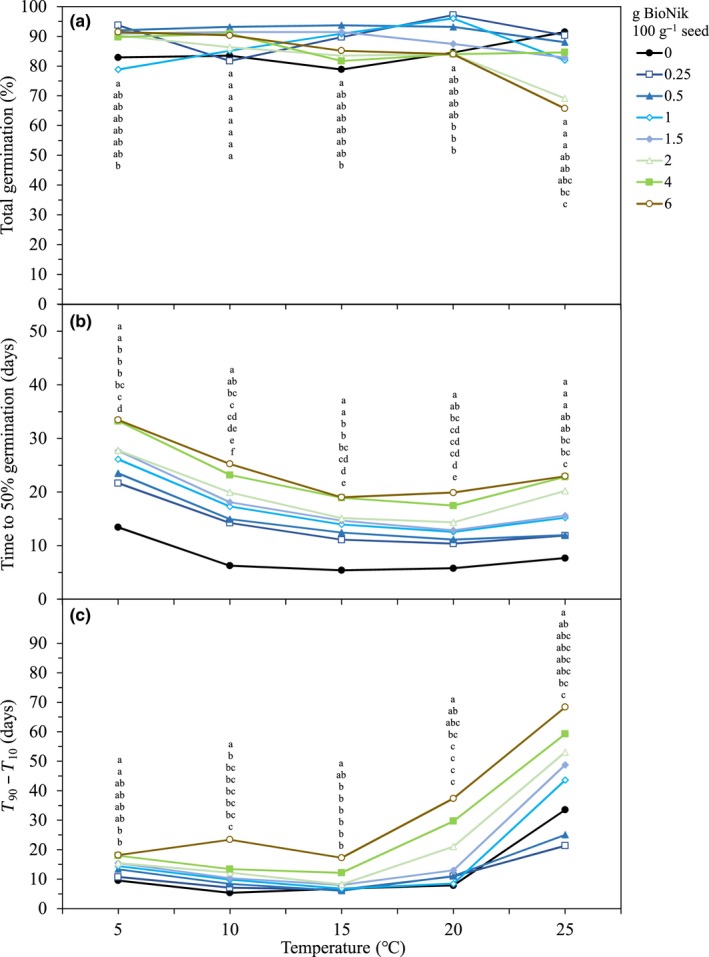
Influence of BioNik (a 25% formulation of S‐abscisic acid) rates applied to bluebunch wheatgrass (*Pseudoroegneria spicata*) seed on (a) final germination percentage, (b) time to reach 50% germination, and (c) germination synchrony at temperatures ranging from 5 to 25°C. Values with the same incubation temperature with different letters are significantly different (*p* < 0.05) at that temperature. Letters correspond with the order of the data points in the figure

Mixed model analysis showed that incubation temperature (*F* = 5.6, *p* < 0.001), ABA dose (*F* = 8.9, *p < *0.001), and the interaction between these two factors (*F* = 8.0, *p* < 0.001) affected final germination percentage. Typically, germination was similar between most treatments at temperatures ranging from 5 to 20°C. Germination for the lower application rates of ABA (0.25–1.0 g BioNik 100 g^−1 ^seed) was slightly higher than untreated seed (Figure 1a). At 25°C, the 2 and 6 g BioNik 100 g^−1 ^seed treatments had lower germination (69% and 65%, respectively) than the other treated and untreated seeds (average of 87%). Across all temperatures, the highest final germination percentage was 97% (0.5 g BioNik 100 g^−1 ^seed, 20°C) and the lowest was 65% (6 g BioNik 100 g^−1 ^seed, 25°C; Figure 1a).

### 3.2 Germination time

Incubation temperature (*F* = 81.8, *p < *0.001) and ABA dose (*F* = 324.38, *p < *0.001) affected T_50_. Typically, as temperature increased, T_50 _values declined to a minimum value at 15°C. At 25°C, T_50_ values began to increase. Within each constant temperature, T_50_ increased with increasing concentration of ABA (Figure 1b). Across all incubation temperatures, there was a strong delay in germination for treated compared to untreated seed (Figure 1b). For example, at 5°C, seed treated with ABA at 0.25, 0.5, 1, 2, 4, and 6 g BioNik 100 g^−1^ seed had T_50_ values that were 8.2, 10.1, 12.7, 14.3, 14.4, 19.8, and 20.0 d longer than untreated seed, respectively (Figure 1b).

### 3.3 Synchrony

Synchrony was affected by incubation temperature (*F* = 90.1, *p* < 0.001), ABA dose (*F* = 109.5, *p* < 0.001), and the interaction between the two (*F* = 10.3, *p < *0.001). Synchrony decreased (i.e. the difference between T_90_‐T_10_ was greater) at 20 and 25°C for ABA doses at or above 2 g BioNik 100 g^‐1^, while all other treatments showed a decrease in synchrony at 25°C (Figure 1c). Within each temperature regime, untreated seed typically had the most synchronous germination, ranging from 5.3 to 33.5 d (Figure 1c). The only exception was at 25°C, where both the 0.25 and 0.5 g BioNik 100 g^−1^ seeds were more synchronous (21.3 and 25.0 d, respectively) than the untreated seeds (33.5 d). Synchrony decreased with increasing ABA dose. For example, at the lowest application rate (0.25 g BioNik 100 g^−1^ seed) synchrony ranged from 6.4 to 21.3 d, while at the highest application rate (6.0 g BioNik 100 g^−1^ seed) synchrony was between 18.1 and 68.3 d, depending on temperature (Figure 1c).

### 3.4 Prediction of seed germination timing in the field

**Figure 2:**
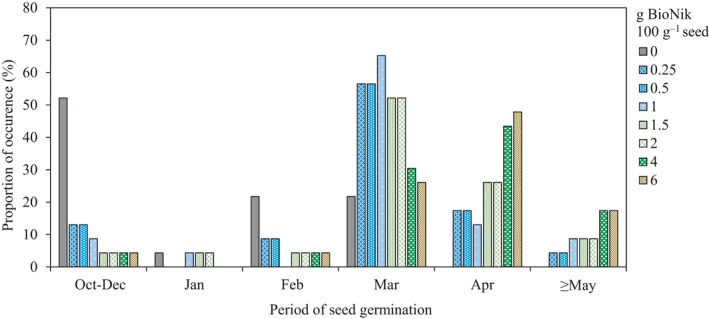
The period of the year when greater than 50% of the seed germinated. Values represent the percentage of occurrence across all sites (four sites) and planting years (6 years) for untreated seed and seed treated with BioNik at rates ranging from 0 to 6 g product  100 g^−1^ seed

All wet‐thermal accumulation models had sufficient accuracy to predict germination time (adjusted *R* ^2^ = 0.51–0.85). For untreated seed, the majority of the seeds were estimated to germinate during the fall and winter (October–February), and only 22% of the time  the majority of the seeds  germinated in March or later (Figure 2). Time required for the majority of the seeds to germinate increased as ABA application rates increased (Figure 2). For almost all sites and planting years, the majority of ABA‐coated seeds were predicted to germinate in spring or early summer (March–May) depending on the application rate of BioNik applied (Figure 2). The majority of seeds coated with 0.25–2.0 g BioNik 100 g^−1^ seed germinated 13%–22% of the time during October–February, 52%–57% of the time in March, and 21%–35% of the time in April or later (Figure 2). Conversely, the majority of seed coated with 4 and 6 g BioNik 100 g^−1^ seed germinated 9% of the time during October–February, 26%–30% of the time in March, and 61%–65% of the time in April or later (Figure 2).

**Figure 3:**
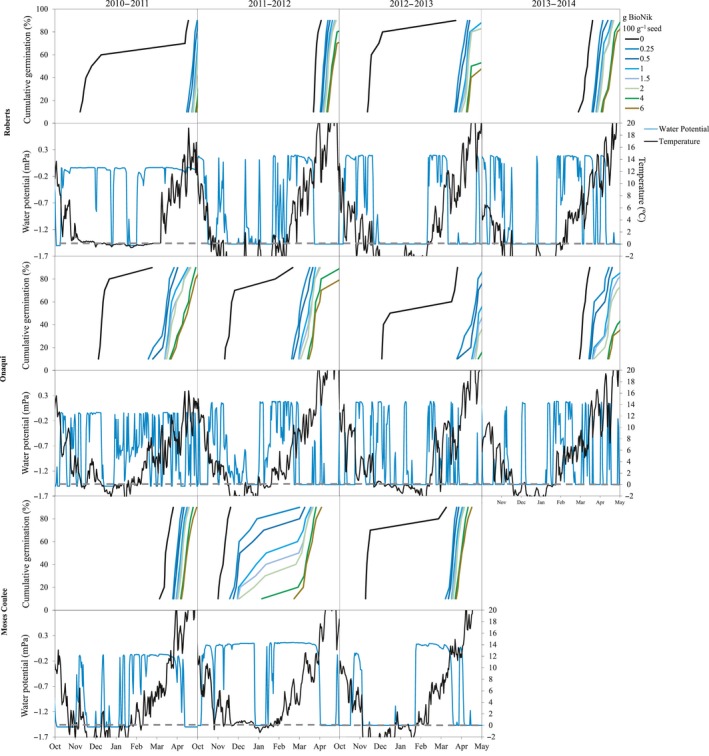
Modeled estimates of the percentage of seeds expected to germinate over time for untreated seed and seed coated with increasing rates of BioNik (ABA), and soil water potential and temperature used to model seed germination timing. Simulations were run with a 15 October planting date on four separate years (2010–2013) for sites in Roberts, ID, Onaqui, UT, and Moses Coulee, WA

**Figure 4:**
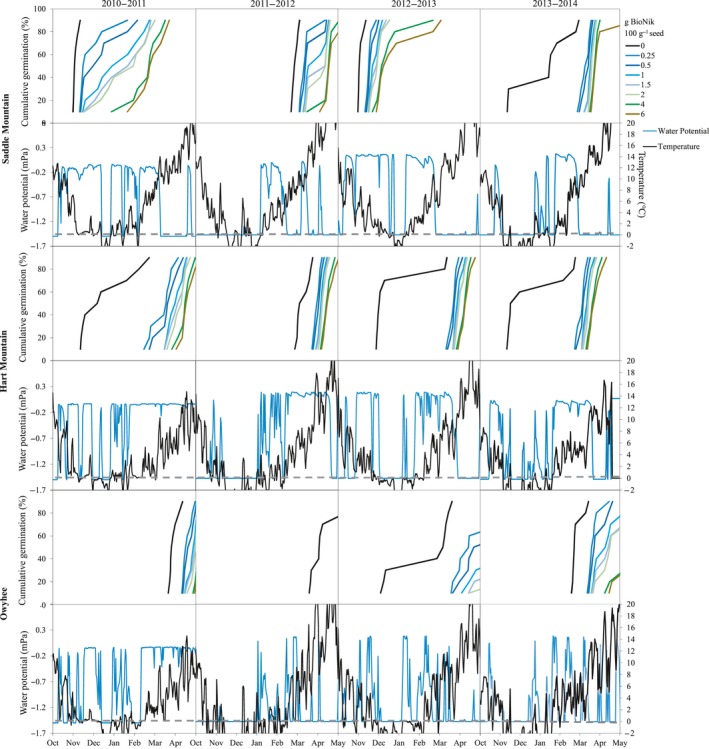
Modeled estimates of the percentage of seeds expected to germinate over time for untreated seed and seed coated with increasing rates of BioNik (ABA), and soil water potential and temperature used to model seed germination timing. Simulations were run with a 15 October planting date on four separate years (2010–2013) for sites in Saddle Mountain, WA, Hart Mountain, OR, and Owyhee, NV

Analysis of individual sites showed high variation in germination timing for the seed treatments by site and year (Figures 3 and 4). While the majority of the time germination of untreated seeds did occur in early fall or winter, when this happened there was commonly a small percentage of seed that would carryover and germinate in spring. In addition to ABA delaying seed germination through winter, application of BioNik spread the period of seed germination at most sites and planting years. The 2012‐2013 simulated planting at Saddle Mountain was the only time when all of the ABA treatments failed to delay seed germination for the majority of the population until spring (Figure 4). The Owyhee site was the only site that did not have at least one year where the majority of the untreated seeds germinated in fall or early winter (Figure 4). At this location, rates above 2 g BioNik 100 g^−1^ seed commonly delayed germination into summer.

## 4 DISCUSSION

Our hypothesis that ABA seed coatings will delay seed germination, with the extent of delay a function of the rate of product applied, was validated through this study. The level of ABA in dormant seeds decreases over time due to after‐ripening (Ali‐Rachedi et al., 2004; Bewley, 1997; Walkersimmons, 1987). Seeds used for restoration projects are usually stored for a year or longer before dispersal. Long storage periods can lead to decreased levels of ABA within the seeds, and quick germination once the seeds are sown. Our study provides a solution to this problem and demonstrates how a BioNik seed treatment application can be tailored to sites where early fall germination may lead to high seedling mortality during winter (Figures 2, 3, 4).

In general, an ABA application had minimal influence on the total number of germinated seeds, except for two coating rates (2 and 6 g BioNik 100 g^−1 ^seed) that showed moderately lower total germination at the highest temperature used in the study (25°C) (Figure 1a). It was our observation that the lower germination for these highcoating rates was due to the seeds spending considerably more time prior to germination in warm moist conditions, which may be more conducive to pathogen attack (Doohan, Brennan, & Cooke, 2003; Koseki & Isobe, 2005). Excessive pathogen attack under the laboratory conditions of this study may not necessarily indicate that ABA would decrease seed viability in the field.

Wet‐thermal accumulation models applied to historic microclimate field data predicted that the majority of untreated seeds germinated within two to three weeks of being planted on most years and sites (Figures 3 and 4). The late fall/early winter germination of untreated seed would subject the seedlings to harsh environmental conditions and potentially result in high seedling mortality (James et al., 2011). Roundy and Madsen (2016) reported that across 14‐sagebrush steppe sites throughout the Great Basin, there was an average of 58 freeze‐thaw periods for the upper 1–3 cm of soil between October and March. Boyd and Lemos (2013) reported a major reduction in emergence and tiller density for bluebunch wheatgrass after exposure to only 4 days of freezing. Our modeling research in combination with Boyd and Lemos’ (2013) physiology studies may indicate that bluebunch wheatgrass and potentially other restoration species are being lost to premature germination over the winter period.

Wet‐thermal accumulation models also showed that an ABA treatment can delay germination of fall plantings until spring (March or later) for most years and sites (Figures 3 and 4), which is anticipated to be a more environmentally favorable condition for seedling survival and plant establishment. Furthermore, increased delay in germination with increasing ABA coating rates suggests that this treatment could be tailored to work on specific sites known to have shorter or longer average freezing periods, or to other species with different germination timing.

While the results of our modeling generally indicated that ABA would allow for germination to occur in spring or later, it is unclear whether ABA seed coatings would improve establishment on all planting years. We assume an early spring germination timing would be most beneficial because this would maximize the period seeds could grow before they were subjected to summer drought (Cline, 2014). Our data indicate that on some sites and years high ABA rates pushed germination to late spring or early summer; under these conditions lower ABA application rates or no ABA (primarily at the Owhyee site) may provide a more optimal germination timing.

Since it may be difficult to predict what the precipitation and temperature will be for a given year, ABA may also be useful in creating a bet‐hedging strategy for seed germination. Varying levels of dormancy within a seed population is a strategy used to mitigate environmental risks in many plants growing in harsh conditions (Hierro et al., 2009; Lewandrowski, Erickson, Dixon, & Stevens, 2016; Venable & Brown, 1988). For species like the one used in this study that typically do not exhibit high levels of seed dormancy, it is probable that the risk of seeding failures could be reduced by using ABA to expand the envelope that seeds germinate under to increase the likelihood that some seeds will germinate within a window that is more favorable for plant establishment and survival.

## 5 CONCLUSION

Current seeding practices need to be altered to enhance success in the sagebrush steppe (James & Svejcar, 2010; James et al., 2011). Many species used in rangeland seeding practices germinate quickly after a fall planting and then are subjected to multiple stressors during winter that can cause mortality, such as freezing, drought, fungal pathogens, and other biotic and abiotic factors (Cline et al., 2018a, 2018b). Modeled estimates of seed germination timing in this study show that the application of BioNik (S‐ABA, Valent BioSciences LLC) has the potential to be a  strategy that overcomes these environmental barriers by delaying seed germination until spring when conditions are more favorable for plant establishment. The application of ABA to seeds may also improve restoration efforts by spreading the period seeds germinate, which may increase the likelihood of seeds germinating during periods of the year with favorable growing conditions. Experiments need to be conducted to verify the results of this research in the field and determine how delaying seed germination with BioNik impacts plant establishment and survival.

## AUTHOR'S CONTRIBUTIONS

MM, TB, and BR conceived the ideas and helped to design the study. WR and DW collected and analyzed the data. WR, MM, BR, SP, PA, and ZA helped to write and organize the manuscript. All authors contributed to the editing of the drafts and gave final approval for publication.

## Data Availability

Data are accessible via the Dryad Digital Repository, https://doi.org/10.5061/dryad.47c8q50.
